# *GhDof1.7*, a Dof Transcription Factor, Plays Positive Regulatory Role under Salinity Stress in Upland Cotton

**DOI:** 10.3390/plants12213740

**Published:** 2023-10-31

**Authors:** Yi Li, Miaomiao Tian, Zhen Feng, Jingjing Zhang, Jianhua Lu, Xiaokang Fu, Liang Ma, Hengling Wei, Hantao Wang

**Affiliations:** 1 Zhengzhou Research Base, National Key Laboratory of Cotton Bio-Breeding and Integrated Utilization, Zhengzhou University, Zhengzhou 450001, China; 2 National Key Laboratory of Cotton Bio-Breeding and Integrated Utilization, Institute of Cotton Research of CAAS, Anyang 455000, China; 3 Western Agricultural Research Center, Chinese Academy of Agricultural Sciences, Changji 831100, China

**Keywords:** upland cotton, DNA-binding with one finger, ABA, H_2_O_2_, salt stress

## Abstract

Salt stress is a major abiotic stressor that can severely limit plant growth, distribution, and crop yield. DNA-binding with one finger (Dof) is a plant-specific transcription factor that plays a crucial role in plant growth, development, and stress response. In this study, the function of a Dof transcription factor, *GhDof1.7*, was investigated in upland cotton. The *GhDof1.7* gene has a coding sequence length of 759 base pairs, encoding 252 amino acids, and is mainly expressed in roots, stems, leaves, and inflorescences. Salt and abscisic acid (ABA) treatments significantly induced the expression of *GhDof1.7*. The presence of *GhDof1.7* in *Arabidopsis* may have resulted in potential improvements in salt tolerance, as suggested by a decrease in H_2_O_2_ content and an increase in catalase (CAT) and superoxide dismutase (SOD) activities. The GhDof1.7 protein was found to interact with GhCAR4 (C2-domain ABA-related 4), and the silencing of either *GhDof1.7* or *GhCAR4* resulted in reduced salt tolerance in cotton plants. These findings demonstrate that *GhDof1.7* plays a crucial role in improving the salt tolerance of upland cotton and provide insight into the regulation of abiotic stress response by Dof transcription factors.

## 1. Introduction

Soil salinization is a major environmental issue worldwide, as studies have indicated that elevated salt levels can impede plant growth and disrupt metabolic processes, ultimately causing a significant decline in crop yield [1]. As salt accumulates in the soil, intracellular water potential surpasses extracellular potential, hindering plant roots’ ability to absorb water and resulting in stunted growth or even plant death due to water scarcity [2]. Furthermore, the accumulation of salt ions can increase reactive oxygen species (ROS) content in plants, leading to the destruction of cell membrane proteins and lipid structures [3]. To cope with salt stress, plants utilize various strategies, such as ion compartmentalization, osmoregulation, scavenging of reactive oxygen species, and regulation of gene transcription [4,5,6]. Transcription factors have the ability to respond to changes in the external environment by regulating their own expression and employing the spatiotemporal specificity of gene expression to activate target genes. In particular, several transcription factor families, such as MYB, NAC, WRKY, and CBF, have been identified as crucial components of the plant’s response to salt stress [7,8,9,10,11].

Dof transcription factors are a plant-specific class of transcription factors that typically consist of 200–400 amino acids [12]. The N-terminal domain of Dof proteins resembles a C2C2-type zinc finger and contains 50–52 conserved amino acid residues that can specifically bind to the core sequence with 5′-AAAG-3′ [13]. The C-terminal transcriptional regulatory domain of Dof transcription factors is highly variable and can interact with a variety of regulatory proteins, enabling the diversity of its function by activating or inhibiting the transcription of genes. Over the years, numerous Dof family members have been discovered and identified in different plants. For instance, 54, 31, 28, and 24 Dof family members were predicted in monocots such as *Zea mays* [14], *Triticum aestivum* [15], *Sorghum bicolor* [16], and *Hordeum vulgare* [17], respectively, while 36, 41, 89, and 78 Dof family members were predicted in dicots such as *Arabidopsis thaliana* [13], *Populus trichocarpa* [18], *Gossypium hirsutum* [19], and *Glycine max* [20], respectively. Previous studies have demonstrated that Dof transcription factors are involved in regulating plant growth [21,22,23,24,25], and more recently, they have been found to play a crucial role in the plant’s response to abiotic stresses [26,27,28,29]. For example, Dof genes in moso bamboo have been shown to be involved in cold, salt, and drought stress responses [30]. In poplars, seven Dof genes responded to osmotic and abscisic acid stress [31]. Additionally, the expression of the tomato Dof transcription factor *TDDF1* can improve the stress tolerance of tomatoes to drought, salt, and various hormones [32]. The Dof transcription factor *SlCDF1-5* in tomatoes is also involved in abiotic stress response [33]. Moreover, the *AtDof5.8* gene binds to specific motifs and activates *ANAC069* [34], thereby responding to salt stress, while the *AtDof1* gene is induced by salt and low-temperature stress, as shown in some studies [35,36]. In summary, the accumulating evidence highlights the critical role of Dof transcription factors in regulating plant growth and development, as well as their crucial involvement in the response to various abiotic stresses.

Cotton (*Gossypium* spp.) is a major cash crop worldwide [37], and upland cotton (*Gossypium hirsutum* L.) is widely grown for its long fibers and high strength. However, the global demand for high-quality cotton is often limited by environmental stresses, especially in Xinjiang, China, which is the country’s main cotton production area and is severely affected by soil salinization [38]. Soil salinization can significantly reduce cotton yield and quality, thereby restricting the sowing area and unit yield of cotton in China. While Dof transcription factors have been shown to play a critical role in plant responses to stress, there are very few reports on Dof genes in cotton, which limits our understanding of their specific roles in cotton. To address the limited understanding of the specific roles of Dof genes in cotton, this study screened and cloned the *GhDof1.7* gene from upland cotton TM-1 and analyzed its function through a series of experiments, including qRT-PCR, GUS staining, subcellular localization, transgenic *Arabidopsis*, VIGS, and yeast two-hybrid and BiFC experiments to study protein interactions. The successful cloning and functional analysis of the *GhDof1.7* gene in this study provides a valuable resource for improving the salt resistance of upland cotton through molecular breeding.

In summary, cotton is a vital cash crop that faces significant challenges due to environmental stresses, such as soil salinization. The identification of Dof transcription factors as key regulators of stress response in cotton provides a potential target for improving cotton’s tolerance to abiotic stressors, which can enhance cotton yield and quality and contribute to global food security. The cloning and functional analysis of the *GhDof1.7* gene in this study enriches our understanding of the role of Dof transcription factors in cotton and provides a valuable gene resource for the molecular improvement of salt resistance in upland cotton.

## 2. Materials and Methods

### 2.1. Gene Cloning and Bioinformatic Analysis

The coding sequence of *GhDof1.7* (*Gh_A10G0541*) and its promoter region sequence of 2000 bp upstream were obtained from the online database CottonFGD (https://cottonfgd.net/, accessed on 1 June 2020) [39]. Specific primers required for amplification were designed using Oligo7.0 software and are listed in Supplementary Table S1. The gene structure was visualized using the online server Gene Structure Display Server (GSDS2.0) (http://gsds.gao-lab.org/, accessed on 20 June 2020). To investigate the evolutionary relationship of *GhDof1.7* with other Dof transcription factors, homologous sequences of AtDof family members were downloaded from the TAIR database (https://www.arabidopsis.org/, accessed on 20 June 2020) and an evolutionary tree was constructed using the neighbor-joining (NJ) method of MEGA7.0 software with a p-distance model and 1000 bootstrap replicates [40]. Multiple sequence alignment of *GhDof1.7* homologs from other species (*Populus tomentosa*, *Glycine max*, *Raphanus sativus*, *Durio zibethinus*, *Hibiscus syriacus*, *Arabidopsis thaliana*, *Theobroma cacao*) was performed using DNAMAN 6.0.3.99 software. The protein structure of GhDof1.7 was analyzed using the Expasy-ProtScale website (https://web.expasy.org/protscale/, accessed on 15 July 2020), and subcellular localization prediction was performed using the online tool Plant-mPloc (http://www.csbio.sjtu.edu.cn/bioinf/plant-multi/#, accessed on 15 July 2020).

### 2.2. Cis-Regulatory Elements Analysis

The promoter region sequence of *GhDof1.7* was analyzed for the presence of cis-regulatory elements using the online tool PlantCARE (http://bioinformatics.psb.ugent.be/webtools/plantcare/html/, accessed on 5 August 2020). The identified cis-regulatory elements were classified based on their functions, including elements involved in plant growth and development, hormone response, and stress response, among others. The results were compared with previous studies to validate the accuracy of the analysis. The identified cis-regulatory elements provide insights into the potential regulatory mechanisms of *GhDof1.7*, and they may contribute to a better understanding of its role in cotton growth and stress response.

### 2.3. DNA and RNA Extraction and qRT-PCR Analysis

Total genomic DNA was extracted using the cetyltrimethylammonium bromide (CTAB) method [41]. Total RNA was isolated from samples using the RNAprep Pure Plant Kit (Tiangen, Beijing, China) according to the manufacturer’s instructions. cDNA was synthesized from RNA using the PrimeScript RT Reagent kit (Takara, Dajin, Japan). Quantitative real-time PCR (qRT-PCR) analysis was conducted using the SYBR Premix Ex Taq (Takara) and the ABI 7500 Real-time PCR system (Applied Biosystems, Shanghai, China). The qRT-PCR primers used in this study are listed in Supplementary Table S2. The qRT-PCR protocol consisted of three steps: step 1, an initial denaturation step at 95 °C for 30 s; step 2, 40 cycles of denaturation at 95 °C for 5 s and annealing/extension at 60 °C for 34 s; and step 3, melting curve analysis. The expression levels of the target genes were normalized to the reference genes *GhActin* and *AtUBQ10* for *G. hirsutum* and *A. thaliana*, respectively. Each sample was analyzed in triplicate, and the data were analyzed using the 2^−ΔΔCt^ formula [42].

### 2.4. Subcellular Localization

The subcellular localization prediction website utilizes the online tool Uniport (https://www.uniprot.org/, accessed on 20 September 2020). The 35S::*GhDof1.7*-GFP vector was constructed and transformed into *Agrobacterium tumefaciens* strain LBA4404. When the transformed *Agrobacterium* reached an OD600 of approximately 1.8–2.0, it was pelleted, resuspended in the infiltration buffer, and incubated in the dark for 3 h before injecting into tobacco leaves (30-day-old seedlings). The infiltrated tobacco leaves were cultured in the dark for 24 h and then observed under a fluorescence microscope to determine the subcellular localization of the *GhDof1.7*-GFP fusion protein. The experiment was repeated at least three times to ensure the reproducibility of the results.

### 2.5. GUS Staining of Transgenic Arabidopsis

The promoter sequence of *GhDof1.7* (2000 bp upstream of the start codon) was cloned into the pBI121-GUS vector. The resulting construct was then transformed into *Arabidopsis thaliana* plants using the floral dip method. Tissue-specific expression of the *GhDof1.7* promoter was analyzed by GUS staining of various tissues, including roots, stems, leaves, inflorescences, and fruit pods, in mature *Arabidopsis* plants (about 30 days). GUS staining was performed using the standard protocol (Jefferson et al., 1987), and the stained tissues were observed and photographed using a stereomicroscope. The experiment was repeated at least three times, each time staining at least 20 plants per line to ensure reproducible results.

The transformation medium was prepared with 0.217 g of 1/2 MS, 3 g of sucrose, and 20 μL of Silwet L-77. Distilled water was added to make up the volume to 100 mL, and the pH was adjusted to 5.7 using sodium hydroxide.

### 2.6. Genetic Transformation of Arabidopsis

The overexpression vector pBI121-*GhDof1.7* was constructed and transformed into *Agrobacterium tumefaciens* strain GV3101. *Arabidopsis thaliana* plants were transformed using the floral dip method [43]. Briefly, flower buds from wild-type *Arabidopsis* were immersed in an *Agrobacterium* suspension for 50 s. The transformed plants were cultured in the dark for 24 h and then transferred to normal growth conditions. Transgenic lines were selected on 1/2 MS medium containing kanamycin to confirm the successful transformation. The experiment was performed once and the obtained results were verified by analyzing multiple independent transgenic lines.

### 2.7. Virus-Induced Gene Silencing (VIGS)

The coding sequences (CDS) of *GhDof1.7* and *GhCAR4* were submitted to the SGN-VIGS website (https://vigs.solgenomics.net/?tdsourcetag=s_pcqq_aiomsg, accessed on 19 March 2021), and 300 bp gene fragments were obtained and cloned into the pYL156 vector. *Agrobacterium tumefaciens* carrying the following plasmids (pYL156-*GhDof1.7*, pYL156-*GhCAR4*, pYL156 (negative control), pYL192 (helper vector), and pYL156-*CAL1* (positive control)) were grown and induced to an OD600 of 1.5. The cells were pelleted, resuspended in the infiltration buffer, and left to stand for 3 h in the dark before injecting into the cotyledons of two-week-old cotton seedlings. The *Agrobacterium* carrying the pYL192 helper plasmid was mixed with other strains in a 1:1 ratio before infiltration. The infiltrated plants were cultured in the dark for 1 day and then transferred to normal growth conditions. The efficiency of gene silencing was assayed in VIGS cotton plants after 20 days of growth, and plants with high gene silencing efficiency were selected for treatment with 200 mM NaCl to observe the phenotype [44]. The experiment was repeated at least three times to ensure the reproducibility of the results.

### 2.8. Plant Materials and Treatment Methods

In this study, cotton plants were grown under normal conditions of 25 °C with a 16-h light/8-h dark cycle, while *Arabidopsis* and tobacco plants were grown under normal conditions of 23 °C with a 16-h light/8-h dark cycle. Uniformly full TM-1 cotton seeds were selected for planting in seedling blocks. When the third true leaves of the plants were expanded, they were treated with various stress conditions, including low temperature (4 °C), 200 mM NaCl, 30% PEG6000, 10 μM ABA, and 2 μM GA (gibberellin). Samples were taken at 0 h, 0.5 h, 1 h, 3 h, 6 h, 9 h, 12 h, and 24 h after treatment, and each sample was repeated three times. All samples were stored at −80 °C for RNA extraction.

Transgenic *Arabidopsis* seeds from the T_3_ generation were germinated on 1/2 MS medium containing 150 mM NaCl, and the germination rate was recorded after 10 days. Wild-type and transgenic *Arabidopsis* plants were treated with a 200 mM NaCl solution at the seedling stage to observe the phenotype. The experiment was repeated at least three times to ensure the reproducibility of the results.

### 2.9. Screening of Yeast Library and Point-to-Point Verification

Initially, we performed self-activation detection in the following groups: self-activation group (pGADT7 + pGBKT7-*GhDof1.7*), positive control group (pGADT7-largeT + pGBKT7-p53), and negative control group (pGADT7-largeT + pGBKT7-laminC). Then, we co-cultured yeast cells containing the pGBKT7-*GhDof1.7* plasmid with a two-hybrid library yeast constructed from salt-treated cotton roots, stems, and leaves. The bacterial broth was incubated for 20–24 h at 30 °C with agitation at 30–50 rpm and then evenly coated on SD/-Trp-Leu-His-Ade (SD-TLHA) medium. After sequencing and aligning the monoclonal yeast, we selected the target gene to construct an AD vector, which was then transferred into Y2HGold yeast together with the pGBKT7-*GhDof1.7* plasmid. Finally, we observed the yeast cells on SD-TLHA/X-gal medium to verify the interaction. The experiment was repeated at least three times to ensure the reliability and reproducibility of the results.

### 2.10. Bimolecular Fluorescence Complementation (BiFC)

The candidate gene CDS sequences were constructed into the pEarleyGate202-YC vector, and *GhDof1.7* was constructed into the pEarleyGate201-YN vector. Ten combinations of *Agrobacterium* were then mixed and injected into tobacco leaves. After 36–48 h of dark incubation, the leaves were observed using laser confocal microscopy. The experimental combinations were as follows:

pEarleyGate201-YN-GhDof1.7 + pEarleyGate202-YC-GhCAR4;

pEarleyGate201-YN-GhDof1.7 + pEarleyGate202-YC;

pEarleyGate201-YN + pEarleyGate202-YC-GhCAR4;

pEarleyGate201-YN-GhDof1.7 + pEarleyGate202-YC-GhSRC2;

pEarleyGate201-YN-GhDof1.7 + pEarleyGate202-YC;

pEarleyGate201-YN + pEarleyGate202-YC-GhSRC2;

pEarleyGate201-YN-GhDof1.7 + pEarleyGate202-YC-GhMKK9;

pEarleyGate201-YN-GhDof1.7 + pEarleyGate202-YC;

pEarleyGate201-YN + pEarleyGate202-YC-GhMKK9;

pEarleyGate201-YN + pEarleyGate202-YC.

The experiment was repeated at least three times to ensure the reproducibility of the results.

### 2.11. Determination of Relevant Physiological Indicators

In this study, we measured the H_2_O_2_ content, SOD (superoxide dismutase) activity, and CAT (catalase) activity using kits provided by Solaibao Biological Company (Beijing, China). The specific operation steps were carried out according to the instructions for use provided by the manufacturer. The experiment was repeated at least three times to ensure the reliability and reproducibility of the results.

## 3. Results

### 3.1. Gene Structure and Sequence Analysis of GhDof1.7

**Figure 1:**
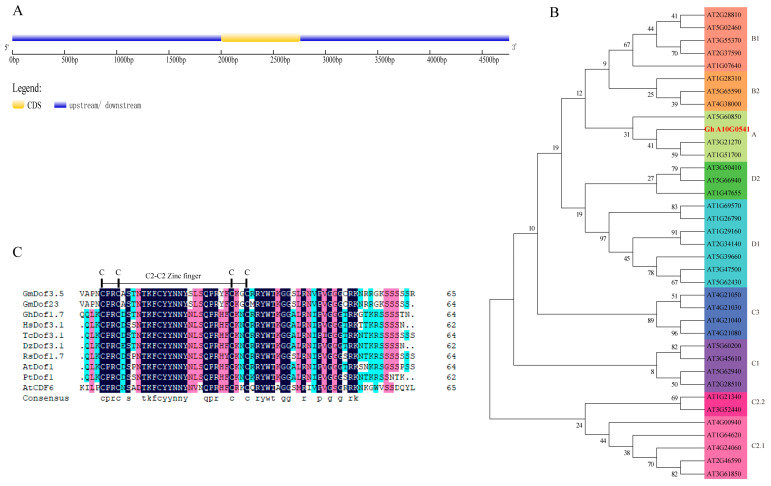
Gene structure, protein sequence, and phylogenetic analysis of *GhDof1.7*. (**A**) Gene structure of *GhDof1.7*. (**B**) Phylogenetic relationship between *Arabidopsis* Dof proteins and GhDof1.7 protein. (**C**) Protein sequence alignment of the GhDof1.7 protein with closely related proteins. The abbreviations before the gene names are as follows: Pt, *Populus tomentosa*; Gm, *Glycine max*; Rs, *Raphanus sativus*; Dz, *Durio zibethinus*; Hs, *Hibiscus syriacus*; At, *Arabidopsis thaliana*; Th, *Theobroma cacao*.

The *AtDof1* has been reported to be involved in various biological processes, including cell cycle regulation, flowering time regulation, stress response, root growth regulation, vascular cell differentiation, and lignin biosynthesis in *Arabidopsis thaliana*. However, the functional characterization of its homologous gene *GhDof1.7* in upland cotton remains unexplored. To investigate the function of the gene, we cloned the *GhDof1.7* gene from upland cotton TM-1. The gene is located on the A10 chromosome and has a genomic DNA length of 4917 bp, with a coding sequence (CDS) length of 759 bp encoding a protein of 252 amino acids. The gene contains one exon and no introns (Figure 1A). The GhDof1.7 protein has a molecular weight (Mw) of 27.62 kDa and an isoelectric point (pI) of 8.64. Sequence alignment analysis revealed that the GhDof1.7 protein shares 52.17% identity with the orthologous protein AtDof1 (AT1G51700) from *Arabidopsis thaliana*. Phylogenetic analysis of the Dof transcription factor family based on the classification of AtDof family [45] divided the family into four main groups (A, B, C, and D) and nine subfamilies. The GhDof1.7 protein was found to be most closely related to group A (Figure 1B). Moreover, comparison of GhDof1.7 with related protein sequences from different species revealed that homologous Dof genes from various species share C2C2 domains (Figure 1C).

### 3.2. Subcellular Localization Results

**Figure 2:**
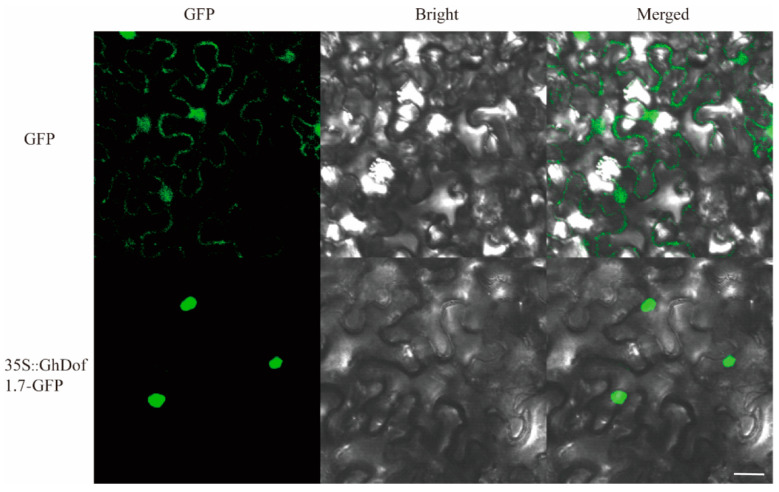
Subcellular localization assays showing that *35S::GhDof1.7*-GFP fusion protein is located in the nucleus. Scale bars, 20 μm. The experiments were replicated at least three times.

The prediction of the online tool Uniport indicated that the protein encoded by the *GhDof1.7* gene is located within the nucleus. To confirm this prediction, we constructed a 35S::*GhDof1.7*-GFP vector and transformed it into *Agrobacterium tumefaciens*, which was then used to infect tobacco leaves. As a control, tobacco leaves were injected with an empty vector. Fluorescence signals were observed in both cell membranes and nuclei in the GFP group, while fluorescence signals were only detected in the nucleus of the *35S::GhDof1.7*-GFP group (Figure 2). These results are consistent with the prediction, indicating that the *GhDof1.7* transcription factor is located in the nucleus and likely plays a role in regulating gene expression in this compartment.

### 3.3. Expression Analysis of GhDof1.7 in Different Tissues

**Figure 3:**
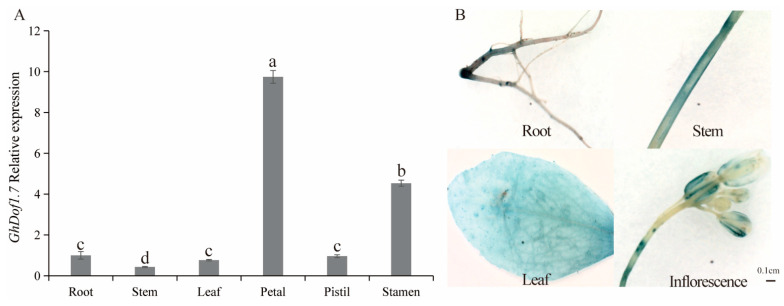
Expression of *GhDof1.7* gene in various tissues of upland cotton. (**A**) Relative expression levels of *GhDof1.7* gene in different tissues. Data are means ± SD (n = 3). Bars followed by different letters indicate significant difference at 5%. (**B**) GUS activity in different tissues of *Arabidopsis thaliana* overexpressing the *GhDof1.7* promoter fragment. Error bars show the standard deviation of three biological repeats. The experiments were replicated at least three times.

To investigate the tissue-specific expression pattern of *GhDof1.7* in upland cotton, we performed qRT-PCR experiments to analyze its relative expression in various tissues. The results showed that the *GhDof1.7* gene had the highest expression level in petals, while in vegetative organs, it was most highly expressed in roots (Figure 3A). To further observe the tissue-specific expression of the *GhDof1.7* gene, we constructed a Pro*GhDof1.7*:GUS vector containing the promoter sequence (2000 bp upstream of the start codon) and transformed it into *Arabidopsis*. GUS staining was performed on different tissues of mature *Arabidopsis*, and GUS activity was detected in the roots, stems, leaves, and inflorescences of *Arabidopsis* with promoter transfer fragments (Figure 3B).

### 3.4. Promoter Sequence Analysis of GhDof1.7

To identify potential cis-acting elements involved in the regulation of *GhDof1.7* gene expression, we extracted the 2000 bp sequence upstream of the start codon (ATG) of this gene for analysis. The results revealed that the cis-acting elements of the *GhDof1.7* gene were mainly classified into three categories: stress-response related elements, hormone-response related elements, and plant growth and development related elements (Supplementary Table S3). The stress-responsive elements included antioxidant response elements (ARE) and low-temperature response elements (LTR); the hormone-responsive elements mainly included abscisic acid response elements (ABRE), gibberellin response elements (P-box), and ethylene response elements (ERE); the growth-related elements included CAT-box and MSA-like elements. These identified cis-acting elements provide insights into the potential regulatory mechanisms of *GhDof1.7* in response to stress and hormone signaling pathways, as well as its role in plant growth and development.

### 3.5. Expression Pattern Analysis of GhDof1.7 under Different Stress Treatments

**Figure 4:**
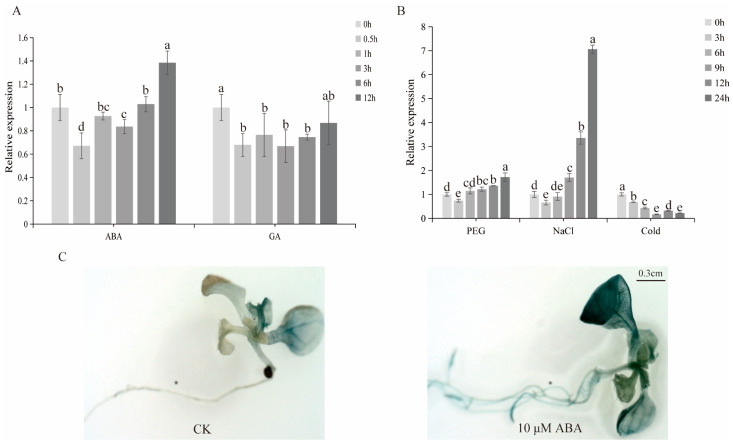
The expression of the *GhDof1.7* gene was regulated by external stress. (**A**) Expression pattern of *GhDof1.7* under ABA and GA stress. Data are means ± SD (n = 3). Bars followed by different letters indicate significant difference at 5%. (**B**) Expression patterns of *GhDof1.7* genes under PEG, NaCl, and cold stress. Data are means ± SD (n = 3). Bars followed by different letters indicate significant difference at 5%. (**C**) GUS activity of *Arabidopsis* overexpressing *GhDof1.7* promoter fragment under ABA stress conditions. The experiments were replicated at least three times.

The promoter sequence analysis of the *GhDof1.7* gene suggested that its expression might be regulated by external stress. To investigate this hypothesis, we treated cotton TM-1 leaves with low temperature, ABA, and GA and cotton roots with salt and drought, and we performed real-time PCR experiments to analyze the expression of *GhDof1.7*. The results showed that the expression of *GhDof1.7* first decreased and then increased under ABA and drought treatments (Figure 4A,B). However, the expression of *GhDof1.7* was down-regulated by GA and cold treatments (Figure 4A,B). Notably, the expression level of *GhDof1.7* was significantly up-regulated after 24 h of salt treatment, reaching its highest level, indicating that this gene is highly induced to be expressed under salt stress (Figure 4B). To further investigate the effect of ABA stress on *GhDof1.7* gene expression, the GUS staining of *ProGhDof1.7::GUS* transgenic seedlings was detected under ABA (10 μM) treatment. The results showed that untreated seedlings displayed weak GUS activity in leaves, while seedlings treated with ABA exhibited stronger GUS activity (Figure 4C). These findings suggest that the expression of the *GhDof1.7* gene is induced by ABA stress treatments, and that it may play a role in regulating the plant’s response to stress.

### 3.6. High Expression of GhDof1.7 Gene Improves Salt Tolerance of Arabidopsis during Germination

**Figure 5:**
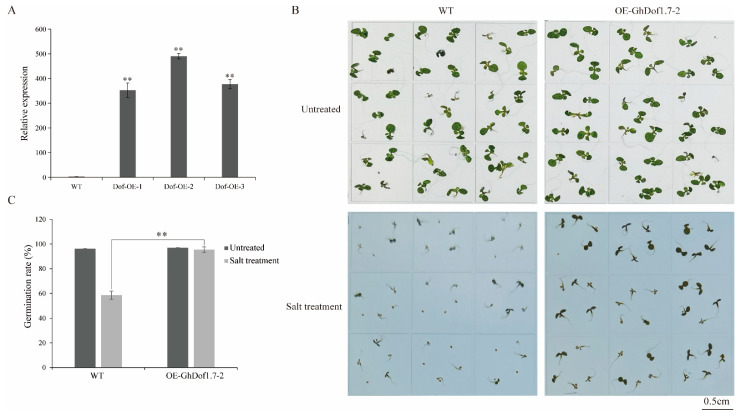
Salt tolerance of transgenic *Arabidopsis* at germination stage. (**A**) Transcript levels of the GhDof1.7 gene in wild-type and transgenic plants; (**B**) germination of wild-type and transgenic plants in 1/2 MS medium and in salt-containing medium; (**C**) germination rates of wild-type and transgenic plants in 1/2 MS medium and in salt-containing medium. Error bars indicate SD (** *p* < 0.01, *n* = 3).

To investigate the potential role of *GhDof1.7* in salt stress resistance, we constructed a 35S::*GhDof1.7* overexpression vector and transformed it into *Arabidopsis*. The expression levels of *GhDof1.7* in wild-type and transgenic *Arabidopsis* were verified by qRT-PCR, and the results showed that the expression of *GhDof1.7* in transgenic *Arabidopsis* was detectable (Figure 5A). We then evaluated the salt tolerance of *GhDof1.7* transgenic *Arabidopsis* at the germination stage by observing their germination rates in 1/2 MS medium and medium containing NaCl. The results showed that the germination rates of wild-type and transgenic *Arabidopsis* in 1/2 MS medium were similar and their growth was comparable. However, the germination rate and growth of *GhDof1.7* transgenic *Arabidopsis* in NaCl-containing medium were significantly better than those of wild-type *Arabidopsis* (Figure 5B,C). These findings suggest that overexpression of *GhDof1.7* can improve the salt tolerance of *Arabidopsis* during germination, highlighting the potential of this gene in improving plant stress tolerance.

### 3.7. GhDof1.7 Promotes Arabidopsis Salt Resistance by Modulating ABA Biosynthesis and Signalling

**Figure 6:**
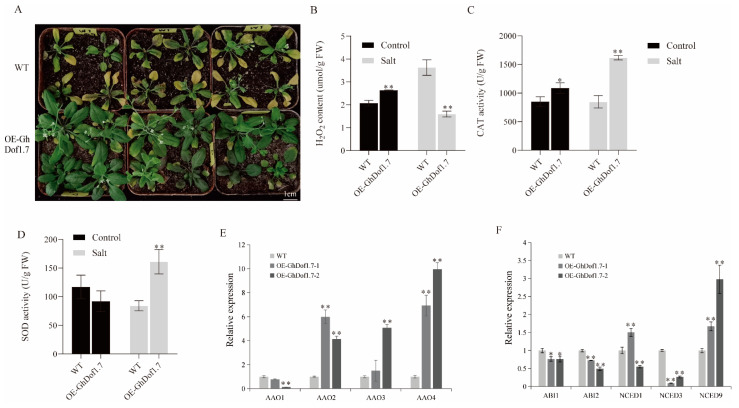
Salt tolerance of transgenic *Arabidopsis* at seedling stage. (**A**) Phenotypes after salt treatment in wild-type and overexpressing GhDof1.7 *Arabidopsis*; (**B**) detection of H_2_O_2_ content in wild-type and transgenic *Arabidopsis* before and after salt treatment; (**C**,**D**) detection of SOD and CAT activity in wild-type and transgenic *Arabidopsis* before and after salt treatment; (**E**,**F**) expression analysis of ABA synthesis and signaling-related genes in wild-type and *GhDof1.7* transgenic *Arabidopsis*. Error bars indicate SD (** *p* < 0.01, 0.01 < * *p* < 0.05, *n* = 3).

To investigate the role of *GhDof1.7* in regulating plant responses to salt stress, we treated wild-type (WT) and transgenic *Arabidopsis* plants overexpressing *GhDof1.7* with 200 mM salt solution and observed their salt tolerance. After 5 days of salt treatment, the leaves of WT turned yellow and the plants were stunted, while *Arabidopsis* transgenic for *GhDof1.7* showed greater adaptability (Figure 6A). We then examined the levels of H_2_O_2_ and the activities of SOD and CAT in wild-type *Arabidopsis* and *Arabidopsis* overexpressing *GhDof1.7* before and after salt treatment. The results showed that H_2_O_2_ content (Figure 6B) and CAT activity (Figure 6C) were significantly increased in *Arabidopsis* plants overexpressing the *GhDof1.7* gene. Under salt stress, H_2_O_2_ content was significantly decreased, and CAT and SOD activities were significantly increased in *Arabidopsis* overexpressing the *GhDof1.7* gene (Figure 6B–D), indicating that *GhDof1.7* overexpression inhibited H_2_O_2_ accumulation and increased the enzyme activities of CAT and SOD in plants under salt stress, thereby enhancing salt tolerance in *Arabidopsis*.

To explore whether *GhDof1.7* transcription factor regulates the expression of ABA-related genes, we performed qRT-PCR experiments to assess the expression changes of ABA biosynthesis and signaling-related genes in transgenic *Arabidopsis* overexpressing *GhDof1.7*. The results showed that the expression levels of ABA biosynthesis and signal transduction-related genes were regulated in *Arabidopsis* plants overexpressing the *GhDof1.7* gene, indicating that *GhDof1.7* can regulate the expression of some ABA biosynthesis genes (*AAO1*-*AAO4*, *NCED2*, *NCED3*, and *NCED9*) and signaling genes (*ABI1* and *ABI2*) (Figure 6E,F). We also identified several putative Dof binding core sequences in the promoter sequences of ABA biosynthesis genes (*AAO1*, *AAO2*, and *AAO4*) (Supplementary Table S4). Therefore, we propose that *GhDof1.7* transcription factor may regulate ABA synthesis by binding to the promoter sequences of genes related to ABA biosynthesis and signal transduction, and then participate in the process of salt stress response. However, further experiments are needed to confirm this hypothesis.

### 3.8. Yeast Two-Hybrid Screening for Interacting Proteins

**Figure 7:**
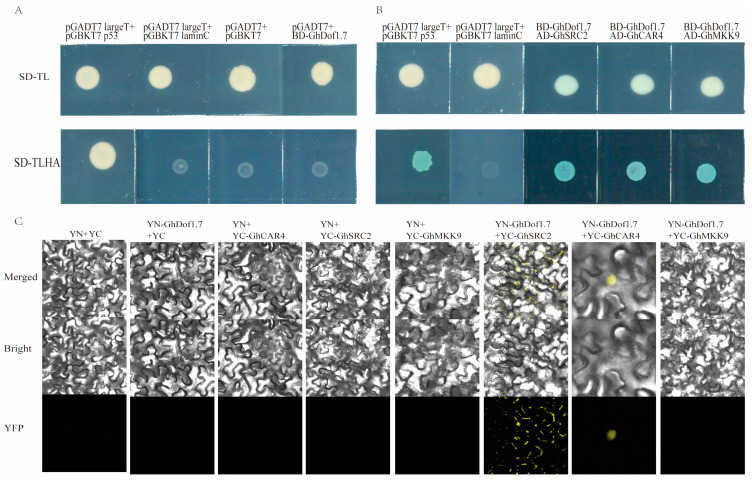
Auto-activation detection of GhDof1.7 in yeast cells and its interaction with candidate proteins. (**A**) Self-activation test result; (**B**) yeast two-hybrid point-to-point validation results; positive control group: pGADT7-largeT + pGBKT7-p53; negative control group: pGADT7-largeT + pGBKT7-laminC; (**C**) BiFC validation mutual interaction results in tobacco.

To identify proteins that interact with GhDof1.7 under salt stress, we screened a protein library using a yeast two-hybrid approach. First, we confirmed that GhDof1.7 does not have transcriptional self-activation activity using a self-activation assay (Figure 7A). We then sequenced monoclonal clones grown on SD-TLHA/X medium and identified a total of 62 potentially interacting proteins after removing duplicates and comparing the results with the TM-1 genome. Functional annotation showed that these genes were mainly associated with signal transduction, growth and development, biotic and abiotic stress, defense response, photosynthesis, and respiration (Supplementary Table S5).

Based on the functional annotation, we selected three target proteins that respond to salt stress (C2-domain ABA-related 4 (GhCAR4), MAP Kinase Kinase 9 (GhMKK9), and Soybean gene regulated by cold-2 (GhSRC2)) for further validation and analysis. We cloned and constructed AD expression vectors for each of these proteins and co-transformed them into Y2HGold yeast competent cells with the constructed pGBKT7-GhDof1.7 plasmid. The transformed cells were identified on SD-TL and SD-TLHA/X plates, and the results showed that all strains grew normally on two-deficient medium (SD-TL), while only the negative control could not grow on four-deficient medium (SD-TLHA) (Figure 7B). This indicates that GhDof1.7 protein may have interacted with GhCAR4, GhMKK9, and GhSRC2 proteins, respectively. To further confirm the reliability of the interaction results, we constructed fusion expression vectors of pEarleyGate201-YN-GhDof1.7, pEarleyGate202-YC-GhCAR4, pEarleyGate202-YC-GhSRC2, and pEarleyGate202-YC-GhMKK9 and transiently expressed them in tobacco leaves. The experimental results showed that YFP yellow fluorescent signal was detected in the combination of YN-GhDof1.7 with YC-GhCAR4, while no fluorescent signal was detected in the combination of YN-GhDof1.7 with YC-GhMKK9 and YC-GhSRC2 experimental combinations. This suggests that GhDof1.7 interacts with GhCAR4, and that the interaction with GhMKK9 and GhSRC2 was a false positive.

### 3.9. GhDof1.7 and GhCAR4 Silencing Reduced Salt Tolerance in Cotton

**Figure 8:**
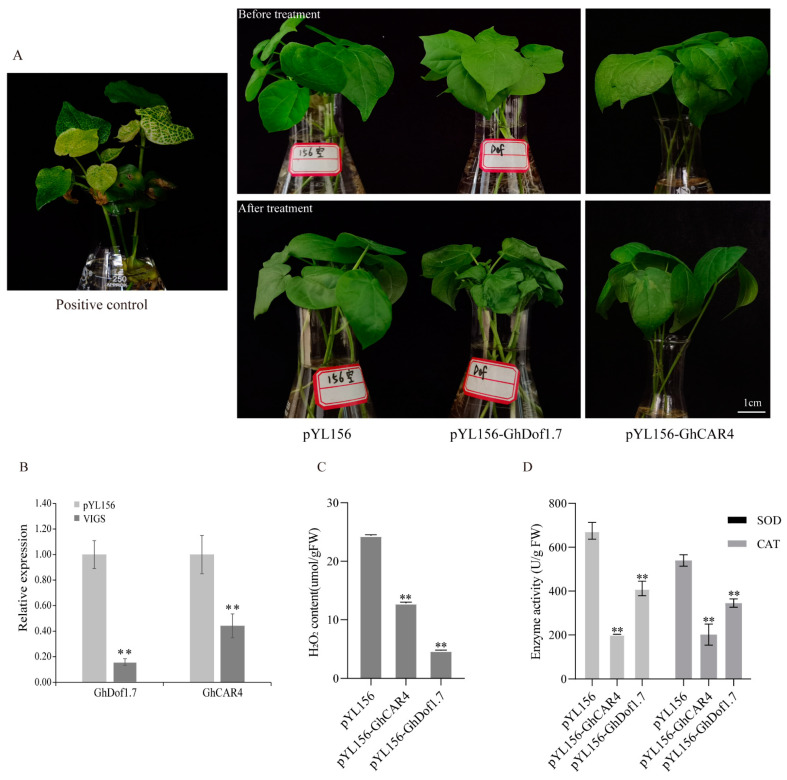
Phenotypic identification and analysis of VIGS cotton. (**A**) Positive control phenotype and phenotypes of plants silenced for *GhCAR4* and *GhDof1.7* under salt stress; (**B**) the expression levels of *GhCAR4* and *GhDof1.7* in pYL156 and VIGS plants; (**C**,**D**) H_2_O_2_ content, SOD activity, and CAT activity in pYL156 and VIGS plants after salt treatment. Error bars indicate SD (** *p* < 0.01, *n* = 3).

*GhDof1.7* plays an important role in the cotton plant’s response to salt stress, and *GhCAR4* is a key interacting protein involved in the regulation of ABA signaling. To further explore the effects of silencing these genes on cotton’s response to salt stress, we constructed VIGS vectors of pYL156-*GhDof1.7* and pYL156-*GhCAR4* and transferred them into cotton cotyledons. When the leaves of positive control plants showed an albino phenotype (Figure 8A), we performed qRT-PCR to detect the expression levels of *GhDof1.7* and *GhCAR4* in control and gene-silenced plants and calculate the silencing efficiency. The results showed that the expression levels of *GhDof1.7* and *GhCAR4* in the gene-silenced plants were significantly lower than those in the empty vector control plants (Figure 8B). We then selected plants with higher silencing efficiency and control plants for salt treatment and observed their response. We found that plants with silenced *GhDof1.7* showed wilting phenotype earlier, and plants with silenced *GhCAR4* showed leaf water loss phenotype earlier compared to control plants (Figure 8A). The results of relevant physiological parameters showed that H_2_O_2_ content, SOD activity, and CAT activity were significantly decreased in pYL156-*GhDof1.7* and pYL156-*GhCAR4* plants compared with pYL156 plants (Figure 8C,D), indicating that silencing the *GhDof1.7* or *GhCAR4* gene reduced the salt tolerance of cotton. These results suggest that both *GhDof1.7* and *GhCAR4* are important regulators of cotton’s response to salt stress, and their silencing can significantly affect the plant’s ability to tolerate salt stress.

## 4. Discussion

### 4.1. GhDof1.7 Gene and Promoter Structure Characteristics

In this study, we cloned the *GhDof1.7* gene from upland cotton TM-1, which has a coding sequence (CDS) of 759 bp encoding 252 amino acids. Subcellular localization and transmembrane structure prediction showed that the GhDof1.7 protein is localized within the nucleus and lacks a transmembrane structure. This localization is consistent with the characteristics of most Dof proteins, which function as transcription factors within the nucleus [46]. The promoter region of the *GhDof1.7* gene contains mainly cis-elements related to abiotic and biotic stresses, photoperiod, growth hormones, and meristem development. Similar cis-elements are also predicted in the promoters of Dof genes in sorghum [47]. The hormone response elements in the promoter region of the *GhDof1.7* gene mainly include abscisic acid (ABA) and gibberellin (GA) response elements. The *AtCDF3* gene in *Arabidopsis* is induced by ABA treatment [33], and the demonstrated role of Dof proteins in gibberellin response in sorghum supports the potential involvement of GhDof1.7 in hormone signaling pathways [47].

### 4.2. GhDof1.7 Is Involved in Cotton Response to Abiotic Stress

Under salt stress, the *OsDof18* transcription factor can regulate the activity of *OsRGLP2* and thereby respond to salt stress [48]. Similarly, eight different *DcDof* genes in carrots were induced to different extents after salt treatment [49]. These findings suggest that Dof transcription factors play important roles in plant response to salt stress. In our study, we observed that the *GhDof1.7* gene in cotton responded most significantly to salt stress. However, the underlying molecular mechanisms of the *GhDof1.7* gene in regulating cotton response to salt stress remain to be fully elucidated. Further studies are needed to explore the detailed regulatory network of *GhDof1.7* in response to salt stress.

Plants accumulate large amounts of reactive oxygen species (ROS) when subjected to salt stress, which disrupts the normal state of the plant. Salt-induced ROS is usually represented by H_2_O_2_ [50,51]. Superoxide dismutase (SOD) is not only a scavenger of superoxide anions but also a major enzyme in the generation of H_2_O_2_ [52]. Catalase (CAT) is primarily responsible for scavenging H_2_O_2_, decomposing it into water and oxygen [53]. When plants encounter salt stress, their SOD activity is enhanced to scavenge oxygen free radicals and generate H_2_O_2_, while the activity of CAT is also enhanced to scavenge H_2_O_2_, thereby maintaining H_2_O_2_ homeostasis in plants. In addition, SOD and CAT are important substances in the antioxidant response [54]. In our study, the expression of the exogenous gene *GhDof1.7* in *Arabidopsis* increased the activities of SOD and CAT under salt stress, which in turn inhibited H_2_O_2_ accumulation in plants. Conversely, cotton leaves with a silenced *GhDof1.7* gene showed an earlier wilting phenomenon, and H_2_O_2_ content and SOD and CAT activities were decreased under salt stress. The molecular signaling cascade mediated by H_2_O_2_ plays a key role in the early response to salt stress in rice and *Arabidopsis* [55]. It has been shown that the Dof transcription factor *CDF4* in *Arabidopsis* can also inhibit H_2_O_2_ secretion by repressing the expression of the catalase2 (CAT2) gene [56]. Thus, we speculate that the *GhDof1.7* gene may regulate the homeostasis of H_2_O_2_ in plants by regulating the activity of the CAT enzyme; however, this conclusion requires further validation.

The ABA signaling system plays an important role in plant responses to abiotic stresses [57]. It has been shown that AAO3 is involved in ABA synthesis under stress conditions [58], and overexpression of *AtNCED3* in transgenic *Arabidopsis* resulted in increased endogenous ABA levels [59]. Moreover, *ABI1* and *ABI2* negatively feedback to regulate the ABA signaling pathway [60]. The Dof transcription factor *CDF4* in *Arabidopsis* can induce *NCED2* and *NCED3* expression, thereby promoting ABA synthesis [56]. After the expression of the exogenous gene *GhDof1.7* in *Arabidopsis*, we observed that ABA synthesis and signaling-related genes were induced or repressed.

### 4.3. GhDof1.7 Interacts with GhCAR4 to Participate in Stress Response

In this study, we screened and validated the GhDof1.7 protein’s interaction with GhCAR4 and GhSRC2 proteins. *GhCAR4* is a C2-domain ABA-related protein involved in regulating ABA signaling [61,62]. Previous studies in *Arabidopsis* and rice have shown that the CAR gene mediates the interaction of PYR/PYL ABA receptors with calcium-dependent proteins, thereby positively regulating ABA signaling [61]. Under salt stress, plants with *GhCAR4* gene silencing exhibited similar phenotypes to plants with *GhDof1.7* gene silencing. Qin et al. [63] showed that *LOT1* affects ABA signaling by regulating *CAR9* expression, thereby regulating plant response to drought stress. Based on these findings, we propose that GhDof1.7 participates in abiotic stress responses by interacting with the GhCAR4 protein to influence ABA signaling.

In summary, our results indicate that GhDof1.7, localized in the nucleus and possessing a promoter region with stress-related cis-elements, interacts with GhCAR4 to potentially modulate ABA signaling and contribute to stress response in cotton.

## Supplementary Materials

The following supporting information can be downloaded at: https://www.mdpi.com/article/10.3390/plants12213740/s1, Table S1: The primers used in vector construction; Table S2: Gene-specific primers used in the qRT-PCR experiments; Table S3: Predicted cis-acting element in the promoter of GhDof1.7; Table S4: 1000 bp sequence upstream of the *Arabidopsis thaliana* AAO1-4 gene.; Table S5: Yeast two-hybrid library screening results.
